# Predictive role of arterial lactate in acute kidney injury associated with off-pump coronary artery bypass grafting

**DOI:** 10.3389/fsurg.2023.1089518

**Published:** 2023-03-16

**Authors:** Ruiming Yu, Tingyi Liang, Longfei Li, Yanwen Bi, Xiangbin Meng

**Affiliations:** ^1^ Department of Cardiovascular Surgery, Qilu Hospital of Shandong University, Jinan, China; ^2^ Department of Endocrinology, Qilu Hospital of Shandong University, Jinan, China; ^3^ Shandong Institute of Medical Device and Pharmaceutical Packaging Inspection, Jinan, China

**Keywords:** acute kidney injury, coronary artery bypass grafting, off-pump, risk factors, arterial lactate, short-term prognosis

## Abstract

**Objectives:**

This observational study aims to explore the predictive role of postoperative arterial lactate in off-pump coronary artery bypass grafting (CABG)-associated acute kidney injury (AKI).

**Materials and methods:**

A total of 500 consecutive patients who underwent off-pump CABG from August 2020 to August 2021 at the Department of Cardiovascular Surgery, Qilu Hospital of Shandong University, were included. Logistic regression analysis was used to confirm the independent risk factors of off-pump CABG-associated AKI. Receiver operating characteristic (ROC) curve was performed to evaluate the discrimination ability and Hosmer–Lemeshow goodness of fit test was performed to evaluate the calibration ability.

**Results:**

The incidence of off-pump CABG-associated AKI was 20.6%. Female gender, preoperative albumin, baseline serum creatinine, 12 h postoperative arterial lactate and duration of mechanical ventilation were independent risk factors. The area under the ROC curve (AUC) of 12 h postoperative arterial lactate for predicting off-pump CABG-associated AKI was 0.756 and the cutoff value was 1.85. The prediction model that incorporated independent risk factors showed reliable predictive ability (AUC = 0.846). Total hospital stay, intensive care unit stay, occurrence of other postoperative complications, and 28-day mortality were all significantly higher in AKI group compared to non-AKI group.

**Conclusion:**

12 h postoperative arterial lactate was a validated predictive biomarker for off-pump CABG-associated AKI. We constructed a predictive model that facilitates the early recognition and management of off-pump CABG-associated AKI.

## 1. Introduction

Acute kidney injury (AKI) is one of the most common postoperative complications in adult cardiac surgery. According to the new Kidney Disease: Improving Global Outcome (KDIGO) consensus criteria (1), the incidence of cardiac surgery-associated acute kidney injury (CSA-AKI) in adults can reach up to 42% (2). Notably, minimal elevations of serum creatinine (SCr) are closely associated with short-term and long-term mortality in patients after cardiac surgery, even for those whose renal function has returned to normal (3–7). Therefore, the early prediction and prevention of CSA-AKI have attracted great attention.

Cardiopulmonary bypass (CPB) is an important independent risk factor for CSA-AKI (8). Renal hypoperfusion, inflammation, and oxidative stress during CPB all increase the risk of CSA-AKI (9–11). Most studies on the risk factors of CSA-AKI have included all types of cardiac surgery without specifically discussing off-pump coronary artery bypass grafting (CABG). Off-pump CABG was developed to reduce the risk of perioperative complications caused by CPB and improve the long-term prognosis of patients (12). However, there is no strong evidence that off-pump CABG is effective in preserving renal function.

Several large-scale studies have found that compared with on-pump CABG, off-pump CABG could indeed reduce the incidence of postoperative AKI, but it did not show better effects on postoperative renal replacement therapy (RRT) and mortality (13–16). This suggests that off-pump CABG may not have significant advantage in improving outcomes in patients with AKI. Considering the wide application of off-pump CABG in cardiac surgery, early recognition and management of AKI in patients undergoing off-pump CABG deserves attention.

Arterial lactate is considered as a biomarker of tissue hypoxia and hypoperfusion. Hyperlactatemia in critically ill patients is closely associated with increased mortality and poor prognosis (17–22). Several studies have found that high lactate level may be linked to the development of CSA-AKI (5, 23–25). However, studies on the relationship between off-pump CABG-associated AKI and lactate are lacking.

The present study aims to explore the predictive role of postoperative arterial lactate in AKI associated with off-pump CABG and construct a predictive model that facilitates the early recognition and management of AKI through a single center retrospective study.

## 2. Materials and methods

### 2.1. Study population

We retrospectively analyzed the patients who underwent off-pump CABG surgery in the Department of Cardiovascular Surgery, Qilu Hospital of Shandong University, from August 2020 to August 2021. Exclusion criteria were patients undergoing on-pump CABG (including intraoperative conversion to CPB), patients undergoing RRT before operation and patients lacking postoperative SCr data. The study has been approved by the Medical Ethics Committee of Qilu Hospital of Shandong University, and patient information was kept anonymously to maintain confidentiality.

### 2.2. Surgical procedures

All off-pump CABG surgeries were performed by experienced cardiac surgeons. Median sternal incision was performed routinely. Left internal mammary artery (LIMA), great saphenous vein, and sometimes radial artery were harvested as vascular grafts. After heparinization, the LIMA was anastomosed to the left anterior descending (LAD) artery, and then anastomosis of aorta to saphenous vein grafts to other stenosed coronary arteries was performed. Anastomotic modes were determined based on the anastomotic site. During the anastomosis, suction stabilizers were used to stabilize the beating heart, and deep pericardial sutures and intra-coronary shunts were routinely used.

### 2.3. Study variables and data analysis

A number of variables were included in this study. Preoperative variables included: age, gender, body mass index, smoking, hypertension, diabetes mellitus (DM), hyperlipemia, history of cerebral disease including cerebral hemorrhage and infarction, chronic obstructive pulmonary disease, previous heart failure, arrhythmia including atrial fibrillation and frequent premature ventricular complexes, percutaneous coronary intervention, New York Heart Association (NYHA) functional classification, recent contrast media exposure (within 7 days before operation), left ventricular ejection fraction (LVEF), laboratory reports, three-vessel coronary heart disease, and left main coronary artery disease. Intraoperative variables included: emergency surgery, operation time, erythrocyte transfusion, urine volume, and fluid replacement. Postoperative variables included: central venous pressure and mean arterial pressure at admission, intra-aortic balloon pump (IABP), low cardiac output syndrome (LCOS), RRT, vasoactive medicine application including dopamine and norepinephrine, duration of mechanical ventilation, erythrocyte transfusion, laboratory reports, complications, length of stay, length of ICU stay, and death. All data were checked twice.

### 2.4. Definition of renal function

We used the 2021 Chronic Kidney Disease Epidemiology Collaboration equation (26) for calculating the estimated glomerular filtration rate (eGFR). AKI was diagnosed and staged according to the KDIGO guidelines published in 2012 (1): increase in SCr ≥ 0.3 mg/dl (≥26.5 μmol/L) within 48 h or increase in SCr to ≥ 1.5 times baseline levels in 7 days or urine volume < 0.5 ml/kg/h for 6 h. Any of the above is diagnosed as AKI. The latest SCr level within 7 days before operation was defined as the baseline, and AKI from ICU admission to discharge were defined as postoperative AKI.

*Severity classification* AKI stage I: increase in SCr ≥ 0.3 mg/dl (≥26.5 μmol/L) within 48 h or increase in SCr to 1.5–1.9 times baseline levels or urine volume < 0.5 ml/kg/h for 6–12 h. AKI stage II: increase in SCr to 2.0–2.9 times baseline levels or urine volume < 0.5 ml/kg/h for ≥12 h. AKI stage III: increase in SCr to ≥4.0 mg/dl (≥353 μmol/L) or increase in SCr to ≥3 times baseline levels or urine volume < 0.3 ml/kg/h for ≥24 h or anuria for 12 h or initiation of RRT.

### 2.5. Statistical analysis

Statistical analysis was performed using IBM SPSS 25.0 and R project software. Continuous variables conforming to normal distribution are expressed as mean ± SD and compared by Student's *t*-test. Non-normally distributed continuous variables are expressed as median and 25th to 75th percentiles and were compared by Mann–Whitney *U*-test. Rank variables were also compared using Mann–Whitney *U*-test. Categorical variables are expressed as frequency and percentage and were analyzed using *χ*^2^ test or Fisher's exact test. *P* < 0.05 (two-side) was considered significant. Logistic regression analysis was performed on the significant variables in univariate analysis to identify independent risk factors. The results of multivariate analysis are presented as odds ratio (OR) and 95% confidence interval (CI). A receiver operating characteristic (ROC) curve was performed to exhibit the predictive ability. The cut-off value was determined when the Youden index was maximum. Hosmer–Lemeshow goodness of fit test was performed to evaluate the calibration of the prediction model. *P* > 0.05 indicated a good calibration ability. Internal validation was performed using the bootstraping method with 1,000 replicates.

## 3. Results

### 3.1. Clinical characteristics of the study population

The study population consisted of 500 patients, including 342 males (68.4%) and 158 females (31.6%). The mean age of the patients was 64 years, and the mean hospital stay was 13 days. The incidence of off-pump CABG-associated AKI was 20.6% (103 patients), including 76.7% (79 patients) with AKI stage I, 7.77% (8 patients) with AKI stage II and 15.5% (16 patients) with AKI stage III. The incidence of postoperative RRT in patients diagnosed with AKI was 7.8%, and 28-day mortality of off-pump CABG was 2.0% (Table 1).

**Table 1:** Clinical characteristics of study population.

Variables	All patients (*n* = 500)	No AKI (*n* = 397)	AKI (*n* = 103)	*P*
Preoperative				
Female	158 (31.6%)	117 (29.5%)	41 (39.8%)	0.044
Age (years)	65 (58, 69)	64 (57, 68)	67 (62, 72)	< 0.01
BMI (kg/m^2^)	25.17 (23.34, 27.34)	25.34 (23.32, 27.38)	24.79 (23.41, 26.67)	0.180
Smoking	218 (43.6%)	176 (44.3%)	42 (40.8%)	0.517
Hypertension	312 (62.4%)	241 (60.7%)	71 (68.9%)	0.125
DM	210 (42.0%)	161 (40.6%)	49 (47.6%)	0.198
DM oral	157 (31.4%)	125 (31.5%)	32 (31.1%)	0.935
DM insulin	53 (10.6%)	36 (9.1%)	17 (16.5%)	0.029
Hyperlipidemia	83 (16.6%)	67 (16.9%)	16 (15.5%)	0.744
History of cerebral diseases	99 (19.8%)	76 (19.1%)	23 (22.3%)	0.470
Arrhythmia	42 (8.4%)	30 (7.6%)	12 (11.7%)	0.182
FVCs	30 (6.0%)	20 (5.0%)	10 (9.7%)	0.075
Atrial fibrillation	18 (3.6%)	14 (3.5%)	4 (3.9%)	0.862
COPD	8 (1.6%)	6 (1.5%)	2 (1.9%)	0.756
Previous heart failure	22 (4.4%)	12 (3.0%)	10 (9.7%)	< 0.01
PCI	65 (13.0%)	52 (13.1%)	13 (12.6%)	0.898
NYHA grade	–	–	–	< 0.01
CM exposure	154 (30.8%)	115 (29.0%)	39 (37.9%)	0.081
Diuretic	103 (20.6%)	69 (18.5%)	34 (26.8%)	0.046
WBC (10^9^/L)	6.37 (5.31, 7.47)	6.33 (5.33, 7.45)	6.51 (5.27, 7.43)	0.528
HGB (g/L)	137 (126, 147)	139 (128, 147)	133 (118, 148)	< 0.01
HCT (%)	41.20 (38.00, 43.60)	41.30 (38.50, 43.70)	39.30 (36.45, 43.45)	< 0.01
PLT (10^9^/L)	226 (190, 271)	226 (190, 266)	231 (195, 286)	0.385
Albumin (g/L)	42.35 (40.15, 44.50)	42.50 (40.50, 44.60)	41.50 (38.70, 43.65)	< 0.01
LDL (mmol/L)	2.02 (1.60, 2.53)	2.02 (1.61, 2.56)	1.96 (1.62, 2.49)	0.711
HDL (mmol/L)	1.00 (0.85, 1.15)	1.01 (0.86, 1.16)	0.99 (0.84, 1.10)	0.158
TG (mmol/L)	1.28 (0.97, 1.72)	1.27 (0.96, 1.69)	1.34 (1.03, 1.88)	0.217
SCr (μmol/L)	76 (64, 86)	73 (64,84)	84 (67. 97)	< 0.01
eGFR (ml/min/1.73 m^2^)	94 (81, 100)	95 (86, 101)	86 (68, 97)	< 0.01
LVEF (%)	60 (51, 65)	60 (52, 65)	57 (44, 64)	0.024
Intraoperative				
3-vessel coronary heart disease	457 (91.3%)	363 (91.4%)	94 (91.3%)	0.955
LM coronary artery disease	91 (18.2%)	71 (17.9%)	20 (19.4%)	0.719
Emergency surgery	34 (6.8%)	23 (5.8%)	11 (10.7%)	0.079
Operation time (min)	270 (240, 300)	265 (240, 300)	290 (261, 310)	< 0.01
Transfusion	195 (39.0%)	146 (36.8%)	49 (47.6%)	0.045
Erythrocyte (U)	0 (0, 2)	0 (0, 2)	0 (0, 4)	< 0.01
Plasma (ml)	0 (0, 0)	0 (0, 0)	0 (0, 0)	0.538
Urine volume (ml)	800 (500, 1000)	800 (500, 1000)	700 (450, 1000)	0.864
Fluid replacement (ml)	2,700 (2500, 3000)	2,700 (2500, 3000)	2,700 (2500, 3425)	0.251
Postoperative				
IABP	51 (10.2%)	20 (5.0%)	31 (30.1%)	< 0.01
RRT	8 (1.6%)	0	8 (7.8%)	< 0.01
LCOS	50 (10.0%)	16 (4.0%)	34 (33.0%)	< 0.01
CVP (cmH_2_O)	8 (6, 10)	8 (6, 10)	8 (7, 2)	0.120
MAP (mmHg)	87 ± 16	87 ± 15	86 ± 17	0.671
Medicine application	182 (36.4%)	121 (30.5%)	61 (59.2%)	< 0.01
Dopamine	161 (32.2%)	105 (26.4%)	56 (54.4%)	< 0.01
Norepinephrine	90 (18.0%)	53 (13.4%)	37 (35.9%)	< 0.01
Mechanical ventilation time (min)	783 (540, 1140)	720 (516, 1026)	1,140 (741, 2490)	< 0.01
0 h artery lactate (mmol/L)	0.9 (0.7, 1.2)	0.9 (0.7, 1.2)	0.9 (0.7, 1.2)	0.824
6 h artery lactate (mmol/L)	2.1 (1.3, 3.4)	1.9 (1.3, 3.1)	2.9 (2.1, 5.4)	< 0.01
12 h artery lactate (mmol/L)	1.6 (1.1, 2.3)	1.5 (1.1, 2.1)	2.5 (1.6, 4.2)	< 0.01
24 h artery lactate (mmol/L)	1.2 (0.9, 1.5)	1.1 (0.8, 1.5)	1.4 (1.0, 1.8)	< 0.01
Transfusion	238 (47.6%)	172 (43.3%)	66 (64.1%)	< 0.01
Erythrocyte (U)	0 (0, 2)	0 (0, 2)	0 (2, 4)	< 0.01
Plasma (ml)	0 (0, 0)	0 (0, 0)	0 (0, 0)	0.068
28-day mortality	10 (2.0%)	1 (0.3%)	9 (8.7%)	< 0.01

AKI, acute kidney injury; BMI, body mass index; CM, contrast media; COPD, chronic obstructive pulmonary disease; CVP, central venous pressure; DM, diabetes mellitus; eGFR, estimated glomerular filtration rate; FVCs, frequent premature ventricular complexes; HCT, hematocrit; HDL, high density lipoprotein; HGB, hemoglobin; IABP, intra-aortic balloon pump; LCOS, low cardiac output syndrome; LDL, low density lipoprotein; LM, left main; LVEF, left ventricular ejection fraction; MAP, mean arterial pressure; NYHA, New York Heart Association; PCI, percutaneous coronary intervention; PLT, platelet; RRT, renal replacement therapy; SCr, Serum creatinine; TG, triglyceride; WBC, white blood cell.

### 3.2. Independent risk factors of off-pump CABG-associated AKI

Hypothesis testing revealed that there were statistical differences between AKI and non-AKI patient groups including age, gender, DM, previous heart failure, NYHA functional classification, preoperative diuretic use, preoperative hemoglobin, preoperative hematocrit, preoperative albumin, baseline SCr, preoperative eGFR, preoperative LVEF, erythrocyte transfusion, IABP application, LCOS, vasoactive medicine application, duration of mechanical ventilation, arterial lactate level after operation (Table 1). In order to more precisely identify risk factors, we categorized the following variables: age, DM, NYHA functional classification, eGFR, LVEF and total erythrocyte transfusion (Table 2).

**Table 2:** Subgroup analysis of Off-pump CABG-associated AKI.

Variables	All patients (*n* = 500)	No AKI (*n* = 397)	AKI (*n* = 103)	*P*
Preoperative				
Age (years)	65 (58, 69)	64 (57, 68)	67 (62, 72)	< 0.01
Age ≥ 70	116 (23.2%)	83 (20.9%)	33 (32.0%)	0.017
60 ≤ Age < 70	242 (48.4%)	192 (48.4%)	50 (48.5%)	0.974
Age < 60	142 (28.4%)	122 (30.7%)	20 (19.4%)	0.023
DM	210 (42.0%)	161 (40.6%)	49 (47.6%)	0.198
Oral pharmacotherapy	157 (31.4%)	125 (31.5%)	32 (31.1%)	0.935
Insulin pharmacotherapy	53 (10.6%)	36 (9.1%)	17 (16.5%)	0.029
NYHA grade	–	–	–	< 0.01
NYHA > 2	178 (35.6%)	127 (32.0%)	51 (49.5%)	< 0.01
eGFR (ml/min/1.73 m^2^)	94 (81, 100)	95 (86, 101)	86 (68, 97)	< 0.01
eGFR > 90	294 (58.8%)	255 (64.2%)	39 (37.9%)	< 0.01
60 ≤ eGFR ≤ 90	180 (36.0%)	134 (33.8%)	46 (44.7%)	0.40
eGFR < 60	26 (5.2%)	8 (2.0%)	18 (17.5%)	< 0.01
LVEF (%)	60 (51, 65)	60 (52, 65)	57 (44, 64)	0.024
LVEF ≥ 60	259 (51.8%)	214 (53.9%)	45 (43.7%)	0.064
50 < LVEF < 60	120 (24.0%)	97 (24.4%)	23 (22.3%)	0.656
LVEF ≤ 50	121 (24.2%)	86 (21.7%)	35 (34.0%)	< 0.01
Intraoperative and postoperative				
Total erythrocyte transfusion (U)	2 (0, 4)	2 (0, 4)	4 (2, 8)	< 0.01
Erythrocyte ≤ 2	297 (59.4%)	260 (65.5%)	37 (35.9%)	< 0.01
2 < Erythrocyte ≤ 4	107 (21.4%)	81 (20.4%)	26 (25.2%)	0.286
Erythrocyte > 4	96 (19.2%)	56 (14.1%)	40 (38.8%)	< 0.01

AKI, acute kidney injury; CABG, coronary artery bypass grafting; DM, diabetes mellitus; eGFR, estimated glomerular filtration rate; LVEF, left ventricular ejection fraction; NYHA, New York Heart Association.

We included the variables that are statistically significant in univariate analysis for logistic regression analysis. The results showed that female gender, baseline SCr level, preoperative albumin level, 12 h postoperative arterial lactate level and duration of mechanical ventilation were independent risk factors of off-pump CABG-associated AKI (Table 3).

**Table 3:** Logistic regression analysis of Off-pump CABG-associated AKI.

Variables	B	*P*	OR	95% CI	
Preoperative					
Female	0.989	0.023	2.690	1.149	6.298
Age ≥ 70 years	0.063	0.853	1.065	0.545	2.081
Insulin therapy for DM	0.581	0.231	1.788	0.691	4.627
Previous heart failure	−0.123	0.874	0.884	0.193	4.045
NYHA grade > 2	0.139	0.693	1.149	0.578	2.285
Diuretic	0.224	0.512	1.251	0.640	2.445
HGB (g/L)	−0.019	0.616	0.981	0.912	1.056
HCT (%)	0.067	0.612	1.069	0.826	1.385
Albumin (g/L)	−0.094	0.039	0.910	0.833	0.995
Baseline SCr (μmol/L)	0.040	<0.01	1.041	1.014	1.068
eGFR < 60 ml/min/1.73 m^2^	0.293	0.707	1.341	0.291	6.174
LVEF ≤ 50%	−0.002	0.996	0.998	0.437	2.282
Intraoperative					
Operation time (min)	0.005	0.129	1.005	0.998	1.012
Postoperative					
IABP	0.368	0.572	1.445	0.403	5.180
LCOS	1.240	0.090	3.457	0.823	14.520
Dopamine	0.316	0.401	1.372	0.656	2.869
Norepinephrine	−0.406	0.401	0.666	0.258	1.719
6-12 arterial lactate (mmol/L)	0.054	0.563	1.056	0.879	1.268
12 h arterial lactate (mmol/L)	0.705	<0.01	2.023	1.461	2.803
24 h arterial lactate (mmol/L)	−0.022	0.933	0.978	0.588	1.627
Mechanical ventilation (min)	0.025	<0.01	1.025	1.007	1.043
Total erythrocyte transfusion > 4 U	0.579	0.123	1.783	0.856	3.718

AKI, acute kidney injury; CABG, coronary artery bypass grafting; DM, diabetes mellitus; eGFR, estimated glomerular filtration rate; HCT, hematocrit; HGB, hemoglobin; IABP, intra-aortic balloon pump; LCOS, low cardiac output syndrome; LVEF, left ventricular ejection fraction; NYHA, New York Heart Association; SCr, serum creatinine.

### 3.3. Arterial lactate level for predicting off-pump CABG-associated AKI

**Figure 1:**
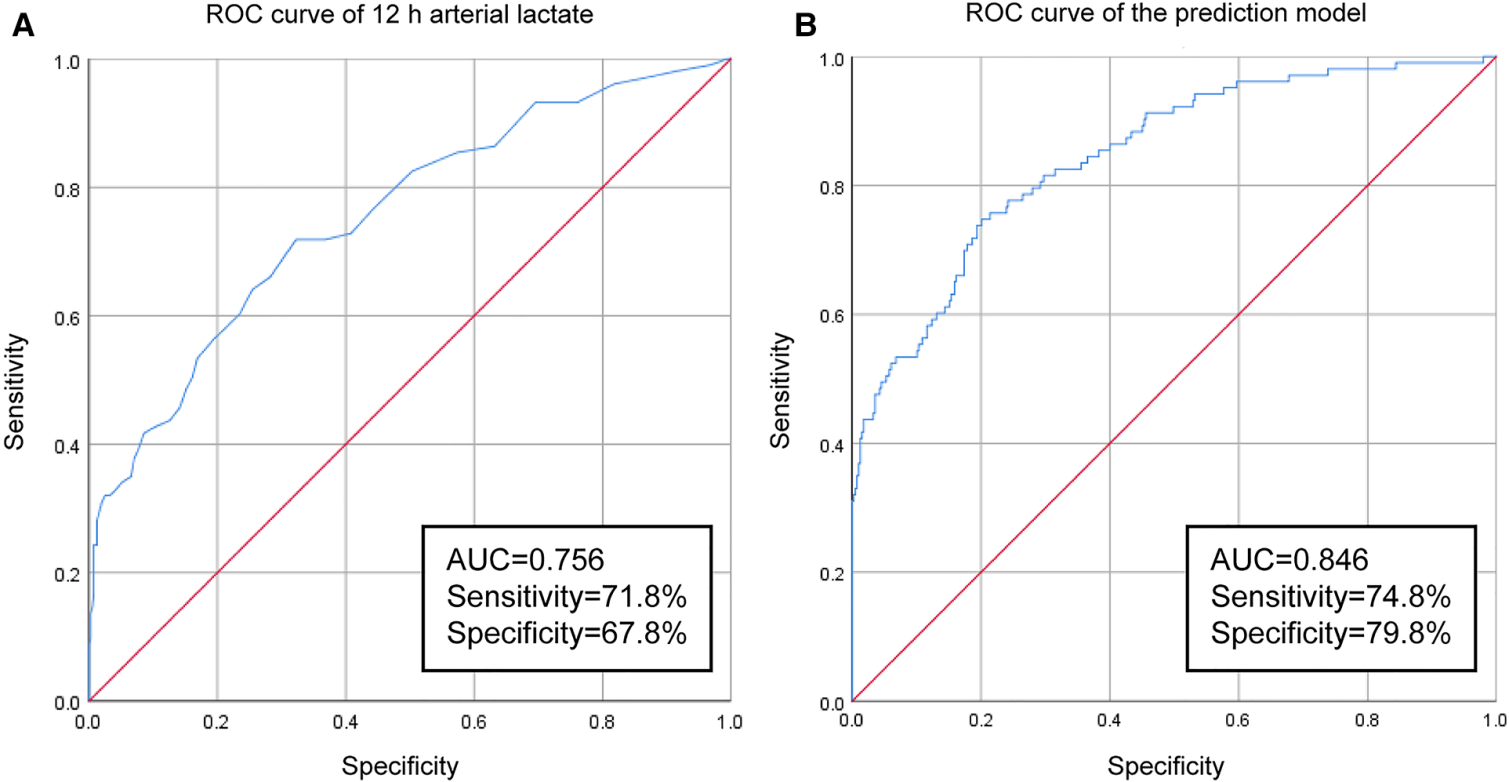
Results of ROC-curve analysis: (A) ROC curve of 12 h arterial lactate for predicting off-pump CABG-associated AKI. (B) ROC curve of the prediction model.

The level of arterial lactate peaked at 6 h after operation. We found that patients in the AKI group had significantly higher arterial lactate levels than patients in the non-AKI group at each postoperative time point, and the difference was the most obvious at 12 h after operation (OR = 2.023, 95% CI = 1.461–2.803). We performed ROC curve analysis to evaluate the ability of 12 h arterial lactate level for predicting off-pump CABG-associated AKI. The area under the ROC curve (AUC) of 12 h arterial lactate level was 0.756 (Figure 1A). The cutoff value was derived to be 1.85 according to the maximum value of the Youden index. The sensitivity was 71.8% and the specificity was 67.8%. The above results confirmed that 12 h arterial lactate after operation is a reliable biomarker for predicting off-pump CABG-associated AKI.

### 3.4. Prediction model of off-pump CABG-associated AKI

**Figure 2.**
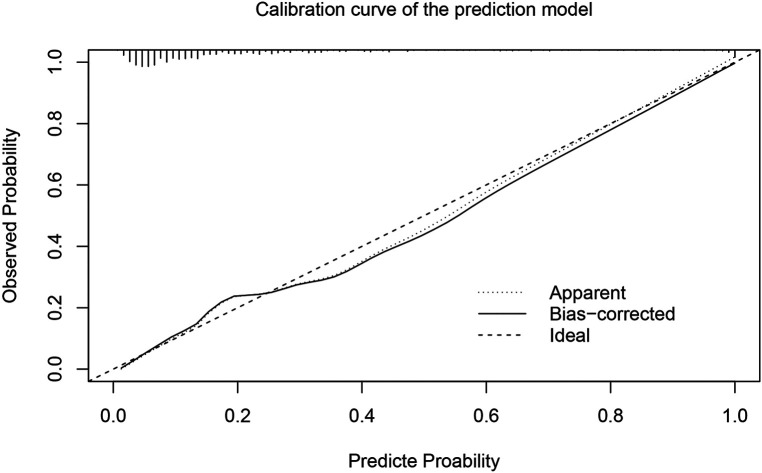
Calibration curve of the prediction model.

In order to more accurately identify high-risk patients with AKI at an early stage, we incorporated the five independent risk factors into a prediction model. The AUC of the new prediction model was 0.846, which exhibited great predictive performance (Figure 1B). The sensitivity was 74.8% and the specificity was 79.8%. The calibrated c-index after internal validation with the bootstrapping method was 0.839. In addition, the result of Hosmer–Lemeshow goodness of fit test confirmed the good calibration ability of the prediction model (*P* = 0.642) and the calibration curve also showed good concordance between predicted and actual probabilities (Figure 2).

### 3.5. Postoperative complications and short-term prognosis

We performed statistical analysis of postoperative complications and 28-day mortality between the AKI group and the non-AKI group. Postoperative LCOS, cerebral infarction, pulmonary infection, incision infection, secondary surgery, pleural effusion, severe respiratory failure, atrial fibrillation and malignant arrhythmia were more frequent in patients with AKI (Table 4). Moreover, patients in the AKI group had longer total hospital and ICU stays, and significantly higher 28-day mortality (Table 4). The above statistical results indicated that patients with off-pump CABG-associated AKI had a worse short-term prognosis.

**Table 4:** Postoperative complications and short-term prognosis.

Variables	All patients (*n* = 500)	No AKI (*n* = 397)	AKI (*n* = 103)	*P* value
Drainage time (d)	7 (6, 9)	7 (6, 8)	8 (6, 11)	<0.01
Cerebral infarction	17 (3.4%)	7 (1.8%)	10 (9.7%)	<0.01
Pulmonary infection	161 (32.2%)	100 (25.2%)	61 (59.2%)	<0.01
Incision infection	5 (1.0%)	1 (0.3%)	4 (3.9%)	<0.01
Multiple operations	7 (1.4%)	3 (0.8%)	4 (3.9%)	0.016
Pleural effusion	143 (28.6%)	97 (24.4%)	46 (44.7%)	<0.01
Severe respiratory failure	45 (9.0%)	12 (3.0%)	33 (32.0%)	<0.01
Atrial fibrillation	119 (23.86%)	75 (18.9%)	44 (42.7%)	<0.01
Malignant arrhythmia	18 3.6%)	4 (1.0%)	14 (13.6%)	<0.01
Length of stay (d)	12 (11, 15)	12 (10, 14)	14 (12, 19)	<0.01
Length of ICU stay (d)	3 (2, 4)	2 (2, 4)	4 (3,7)	<0.01
28-day mortality	10 (2.0%)	1 (0.3%)	9 (8.7%)	<0.01

## 4. Discussion

The development of AKI during hospitalization significantly increases the risk of subsequent chronic kidney diseases and death (27). Second only to sepsis, patients undergoing cardiac surgery are more likely to develop AKI because of hemodynamic instability, CPB, and increased inflammatory response (28). Therefore, research on risk factors of CSA-AKI has attracted much attention and many predictive models have been developed for this purpose.

Due to the lack of consensus on the definition of AKI, the initial predictive models, such as the Cleveland Clinic score, the Mehta score, and the Simplified Renal Index, were mainly targeted at severe AKI requiring RTT (29–31). Later on, according to the definition of AKI established by the KDIGO guidelines, many researchers have developed predictive models for CSA-AKI at all stages (32, 33). However, the mechanisms underlying AKI after off-pump CABG differ from on-pump cardiac surgery. During CPB, hypothermia, hypoperfusion, non pulsatile blood flow, systemic inflammatory response, vasoactive agent, acidosis, intravascular haemolysis, etc., all lead to renal vasoconstriction and renal hypoperfusion (34–36). Ischemia reperfusion injury following CPB further contributes to renal impairment (2). Off-pump CABG may avoid risk factors associated with CPB that cause renal injury. To the best of our knowledge, there are few published studies addressing the risk factors of off-pump CABG-associated AKI (37–44). Our study revealed the predictive role of 12-h postoperative arterial lactate in off-pump CABG-associated AKI and provided a new prediction model for the early recognition and management.

Lactate is an important biomarker to evaluate and monitor tissue perfusion in critically ill patients. High arterial lactate level can reflect the imbalance of tissue oxygen metabolism which contribute to the development of AKI. In the study by Lopez-Delgado and coworkers, 24-h postoperative arterial lactate was an independent risk factor of CSA-AKI (5). Zhang and colleagues found that normalized lactate load was independently associated with CSA-AKI (23). A prospective trial involving 100 low-risk patients showed that postoperative lactate was a reliable predictor of CSA-AKI (45). The above studies have demonstrated a correlation between postoperative lactate and CSA-AKI. However, they only included cardiac surgery with CPB but not involving off-pump CABG. Our study found that the arterial lactate levels at 6 h, 12 h and 24 h after off-pump CABG in the AKI group were significantly higher than that in the non-AKI group. And we confirmed that 12 h arterial lactate was a potent predictor of off-pump CABG-associated AKI, which has not been reported in other studies.

Arterial lactate is a rapidly available indicator. Both circulating hypovolemia and microcirculatory derangement may lead to tissue hypoperfusion, which causes tissue hypoxia and increases lactate levels. Persistent high lactate levels are closely associated with organ failure (46, 47). In addition, elevated lactate levels may be a consequence of AKI. Legouis and colleagues proposed that changes in renal gluconeogenesis during AKI may increase mortality by affecting systemic metabolism (48). Their study found that the level of blood lactate in mice exposed to renal ischemia-reperfusion injury increased significantly and the clearance rate of lactate decreased significantly. Patients with AKI are more likely to suffer from impaired metabolism (low to normal glucose/high lactate), which is closely related to increased mortality. And in their study, thiamine supplementation can reduce mortality in patients with AKI by increasing lactate clearance and glucose production.

Our study focused more on the predictive value of lactate levels at early time points. Preoperative and intraoperative predictors were very timely, but not accurate enough. Predictors during late postoperative period are accurate, but not timely. The 12 h postoperative arterial lactate is both timely and reliable in predicting off-pump CABG-associated AKI. And we constructed a prediction model with good discrimination and calibration by combining the other four independent risk factors. There are several well-known risk factors for AKI, such as hypertension, DM, erythrocyte transfusion, LCOS, etc., which were significantly different between AKI and non-AKI group. However, they were not independent risk factors in the multivariate analysis, which may be due to dilution by other risk factors.

Preoperative albumin was included in the prediction model as an independent risk factor (OR = 0.910, 95% CI = 0.833–0.995). The association of albumin and AKI has been discussed mainly in patients with liver dysfunction. Albumin infusion can effectively improve circulating volume and decrease inflammatory response (49, 50). The combination of albumin and vasoconstrictors is the most important treatment for hepatorenal syndrome and can effectively improve the renal function (51, 52). In the study by Lee and colleagues, preoperative serum albumin levels <4.0 g/L was an independent risk factor for off-pump CABG-associated AKI (OR = 1.83, 95% CI = 1.27–2.64), and administration of 20% exogenous albumin before operation was able to reduce the risk of AKI (40, 53). Our study also confirmed the association. However, more studies are needed on whether albumin supplementation can prevent CSA-AKI.

Statistical analysis of short-time outcome after off-pump CABG indicated a higher incidence of postoperative complications and prolonged hospital stay among patients in the AKI group, which convinced us that the impact of off-pump CABG-associated AKI cannot be overlooked.

There are two limitations in our study. First, as a single center retrospective study, the findings were not validated in an external study population. Second, our study addressed only in-hospital complications and short-term survival but did not perform follow-up on long-term prognosis of patients. In the future, we would like to strengthen the management of patients based on the risk factors, such as albumin expanding volume therapy or thiamine improving glucose metabolism, to find out which strategy is able to prevent off-pump CABG-associated AKI.

## 5. Conclusion

Our study demonstrated the predictive value of 12 h postoperative arterial lactate in off-pump CABG-associated AKI and constructed a reliable prediction model which contributes to the early recognition and management of off-pump CABG-associated AKI.

## Data Availability

The original contributions presented in the study are included in the article/Supplementary Material, further inquiries can be directed to the corresponding author.
